# Improving the quality of XAFS data

**DOI:** 10.1107/S1600577518006021

**Published:** 2018-05-29

**Authors:** Hitoshi Abe, Giuliana Aquilanti, Roberto Boada, Bruce Bunker, Pieter Glatzel, Maarten Nachtegaal, Sakura Pascarelli

**Affiliations:** a High Energy Accelerator Research Organization (KEK), Tsukuba, Japan; b Elettra-Sincrotrone Trieste, Basovizza, Italy; c Diamond Light Source, Oxfordshire, UK; d University of Notre Dame, Notre Dame, IN, USA; e European Synchrotron Radiation Facility, Grenoble, France; f Paul Scherrer Institute, Villigen, Switzerland

**Keywords:** XAFS, quality of XAFS data, sources of noise in XAFS, harmonic rejection in XAFS, good practice in XAFS experiments

## Abstract

Following the Q2XAFS Workshop and Satellite to IUCr Congress 2017 on ‘Data Acquisition, Treatment, Storage – quality assurance in XAFS spectroscopy’, a summary is given of the discussion on different aspects of a XAFS experiment that affect data quality. Some pertinent problems ranging from sources and minimization of noise to harmonic contamination and uncompensated monochromator glitches were addressed. Also, an overview is given of the major limitations and pitfalls of a selection of related methods, such as photon-out spectroscopies and energy-dispersive XAFS, and of increasingly common applications, namely studies at high pressure, and time-resolved investigations of catalysts *in operando*. Advice on how to avoid or deal with these problems and a few good practice recommendations are reported, including how to correctly report results.

## Abstract 2

A summary of the discussion on aspects of a XAFS experiment that affect data quality, held at the Q2XAFS Workshop Satellite to IUCr Congress 2017 on ‘Data Acquisition, Treatment, Storage – quality assurance in XAFS spectroscopy’ is reported.

## 1. Introduction   

The XAFS (X-ray absorption fine-structure) community has been constantly growing since the first quantitative work exploiting synchrotron radiation, almost 50 years ago. We have seen a gradual transition in the nature of this community, that started with a few scientists, mainly physicists (Sayers *et al.*, 1971 ▸; Lee *et al.*, 1981 ▸), who developed the method, and evolved to include thousands of experts in a plethora of different scientific areas, including biology, geology, chemistry, cultural heritage and many more, often with little or no academic background in physics. In parallel, XAFS methods have also evolved, triggered by facility scientists developing their in-house research programmes, while accelerator-based sources developed into higher-brilliance third-generation and diffraction-limited storage rings and free-electron lasers. Today we see more complex experimental setups aimed at a more exhaustive investigation of systems, including more relevant composition, space and time resolution, under a variety of external stimuli and adopting new XAFS-based methods based on photon-out spectroscopies. Many of these developments are accompanied by larger data sets and more complex data reduction protocols. In parallel, XAFS is becoming an increasingly popular characterization technique, used more and more by non-experts. XAFS is also becoming available on the laboratory scale, providing easier access to this method by non-expert users (Seidler *et al.*, 2014 ▸; Padamati *et al.*, 2017 ▸). To maintain modern XAFS applications accessible to the larger user community without compromising the accuracy of the information, it is important to make all aware of the outstanding issues and to continue efforts toward experimental standardization. For a review on the history of the efforts devoted to establishing XAFS standards and criteria, see, for example, Oyanagi & Stern (2017 ▸).

It is in this spirit that a session of the Q2XAFS^1^ Workshop Satellite to IUCr Congress 2017 was devoted to a discussion on the outstanding and unsolved problems affecting XAFS data quality. Issues closely related to beamline optics and detection were discussed at large, with focus on sources and minimization of noise, harmonic contamination and uncompensated monochromator glitches. This discussion is summarized in §2. In §3 we report some issues that were addressed on the limitations and difficulties affecting a selection of XAFS-related methods, and in particular energy-dispersive XAS and photon-out spectroscopies. Good practice recommendations are reported here, including how to correctly report results. Finally, in §4 we focus on two increasingly common XAFS applications at high-brilliance sources, *i.e.* high-pressure studies and time-resolved investigations of catalysts. We report on recent developments and offer some advice on how to avoid or deal with inherent difficulties. There are of course other possible sources of error that were not discussed at the workshop. For example:

(i) Sample thickness and homogeneity and effects on the amplitude of XAFS (Goulon *et al.*, 1982 ▸).

(ii) Non-linearity of detectors when working at the high-flux condition: ionization chambers (Pettifer *et al.*, 1999 ▸), fluorescence detectors (Woicik *et al.*, 2010 ▸; Walko *et al.*, 2011 ▸).

(iii) Radiation damage and associated spectral changes (Holton, 2007 ▸; Bertrand *et al.*, 2015 ▸).

(iv) Experimental challenges with dilute systems and complex molecules (Chantler *et al.*, 2012*a* ▸).

Many of these issues and how to deal with them are addressed in the comprehensive paper by Chantler *et al.* (2012*b* ▸), an outcome of the 2011 Q2XAFS Workshop. Finally, the book by G. Bunker (Bunker, 2010 ▸) also provides, in ch. 3, practical issues of sample preparation, experimental methodology, choice of detectors, sources of measurement errors and measures of how to avoid them.

## 2. Sources of errors related to beamline optics and detection   

### 2.1. Sources and minimization of noise   

‘Noise’ in XAFS includes contributions from (i) stochastic noise, *e.g.* counting statistics, (ii) electronic noise, both analog and digital, (iii) X-ray beam instability from the X-ray source, and (iv) mechanical motion of optical elements or sample. In terms of data quality, it is useful to generalize this to include other artefacts such as slowly varying thermal fluctuations in monochromators, mirrors, *etc*., sample issues, *e.g.* inhomogeneity, crystallinity, *etc*., and other monochromator or mirror issues.

Stochastic noise, *e.g.* counting statistics, is generally only a problem in low-count-rate and/or high-background situations, *e.g.* photon-out spectroscopies such as dilute fluorescence, inelastic scattering, *etc*. Note that even with high absolute count rates, scattered or fluorescence background may dominate and the actual ‘signal’ count rate might be relatively low. In these common cases, the statistical fluctuation in background counts generally dominates over fluctuations in signal photon count rate. This has recently been covered in detail in explorations of the limits of fluorescence detection at high-flux beamlines (Heald, 2015 ▸).

To optimize the signal-to-noise ratio [S/N = 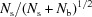 where 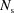 and 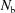 are the counts of the signal and of the background, respectively], one wants to reduce the background signal, but generally the signal will be reduced by these measures as well. This can be accomplished by the use of X-ray filters and Soller slits, photon-counting detectors (*e.g.* Si or Ge solid state detectors) allowing energy discrimination, or the use of crystal or multilayer X-ray analyzers. Each of these approaches has its own strengths and limitations (see, for example, Bunker, 2010 ▸; Heald, 2015 ▸).

Electronic noise or artefacts can also contaminate the signal, but can generally be minimized by improving shielding of detectors and pre-amplifiers and eliminating ground loops due to shared signal paths. Another source of error includes pulse-counting issues such as pulse pile-up, dead-time corrections, or analyser window shifts for high count rates.

Transmission data can also be susceptible to noise if the ‘dark current’ offset voltage is improperly calibrated. This offset is necessary to avoid low-current nonlinearity in voltage-to-frequency converters, but, if not removed after voltage-to-frequency conversion, will introduce nonlinearities of its own as intensity fluctuations will not be correctly normalized.

X-ray beam instability from the X-ray source is an additional source of error, affecting data quality. Insertion devices might cause issues more often than bending-magnet sources. For instance, the high heat load from wigglers can cause thermal stability issues with beamline components, while the peaked spectrum from undulators either require scanning the undulator gap (possibly increasing noise) or, if the gap is tapered to broaden the spectrum, spatial structure can be introduced in the beam that can also introduce artefacts as a function of energy.

Mechanical stability is very important to minimize point-to-point intensity fluctuations, and thermal stability essential to avoid long-term data distortion. Additionally, monochromator ‘glitches’, *i.e.* spurious reflections from monochromator crystals, can cause unwanted changes in intensity, beam direction, harmonic content or polarization. This can depend strongly on sample details with inhomogeneous samples much more sensitive to these artefacts. While some care in sample preparation can help, sometimes the only real solution is to use a different monochromator crystal set with different φ orientation. Many beamlines have glitch spectra mapped out for users (Stanford Synchrotron Radiation Lightsource, 1999 ▸).

A very important issue which is often underestimated is detector linearity. Intensity fluctuations due to source position/angle or X-ray optics would ideally cancel out if both *I*_s_ and incident *I*_0_ are measured and then the ratio taken. If the response is nonlinear due to detectors, electronics, harmonic contamination or sample issues, fluctuations will not cancel resulting in increased noise or other artefacts. This is important to minimize in beamline design, beamline configuration for a particular experiment, and experimental setup by users. This is important in transmission as well as fluorescence measurements, and for ion chambers can be exacerbated by ion-chamber recombination for very intense X-ray beams.

### 2.2. Contamination from harmonics   

One of the important factors to determine the quality of XAFS spectra is how to handle and reject harmonics of the primary X-ray beam (Bonse *et al.*, 1976 ▸; Sainctavit *et al.*, 1988 ▸). The primary X-ray beam used for measurements is usually monochromated by double-crystal monochromators (DCMs) of Si(111) or Si(311), and the higher harmonics should be rejected by mirrors. Harmonic contamination in the X-ray beam can produce distortions in the XAFS spectrum that can be misinterpreted, yielding incorrect results.

**Figure 1:**
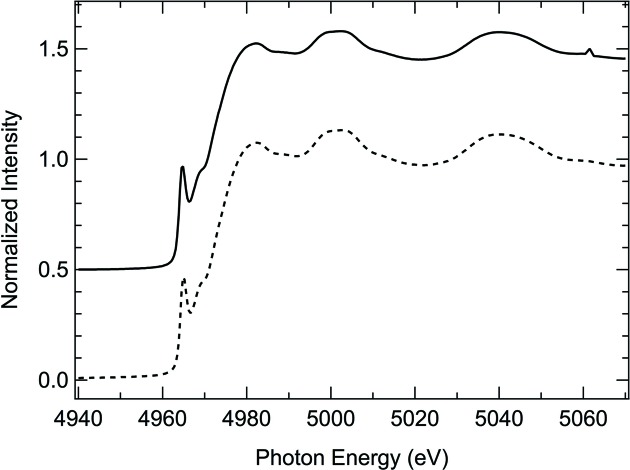
Ti foil *K*-edge XANES spectra. Dashed and solid lines correspond to data measured with a 50% detuning and a fully tuned DCM. The spike at ∼5060 eV present in the solid line XANES can be attributed to lack of normalization of a monochromator glitch, due to insufficient harmonic rejection.

It is not easy to become aware of harmonic contaminations in EXAFS of samples that are being studied, while it is relatively easy to notice those of standard materials such as commercially available metal foils and oxide powders. One simple way is to examine the XAFS spectra carefully. If some reproducible non-statistical noise, or spike feature, is found in the spectra, this could be due to a lack of proper *I*_0_ normalization (*i.e.* monochromator glitches or other features present in the energy dependence of the *I*_0_ are not fully normalized out) and non-efficient harmonics rejection should be suspected. Fig. 1 ▸ illustrates the XANES of the spectra recorded on a reference Ti foil under different experimental conditions. The dashed line is measured using a detuning of 50% of the DCM, while the solid line on the other hand is measured using a fully tuned DCM. The comparison shows that in the case of a fully tuned DCM a spike is visible at ∼5060 eV, caused by the lack of normalization of a monochromator glitch. In the EXAFS regime, oscillations are small and harmonic contamination, particularly in the high-*k* region, can seriously damage data analysis.

**Figure 2:**
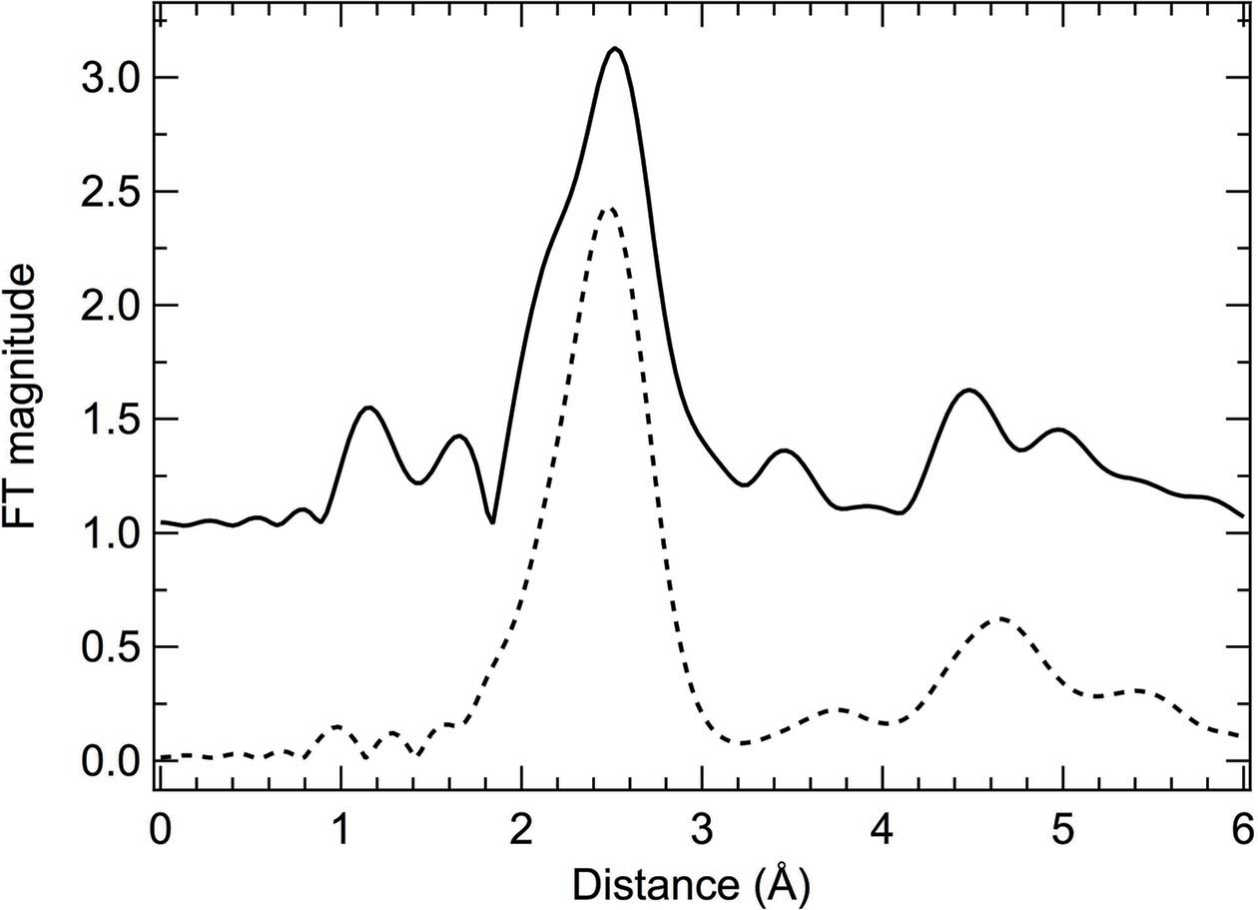
Ti foil *K*-edge EXAFS FTs. Dashed and solid lines correspond to data measured with a 50% detuning and a fully tuned DCM. The ‘ghost shoulder’ at ∼2.2 Å in the solid line spectrum cannot be reproduced by a single Ti–Ti path..

The Fourier transforms (FTs) of Ti *K*-edge EXAFS on Ti foil are shown in Fig. 2 ▸. In the 50% detuned case (dashed line), the peak at *R* ≃ 2.5 Å in the FT can be well reproduced using a single Ti–Ti path, with an *R*-factor of 0.009. However, in the fully tuned case (solid line), besides the presence of an unphysical signal between 1 and 1.8 Å, a ghost shoulder structure is present around 2.2 Å and the peak can no longer be correctly fitted by a single Ti–Ti path.

This example illustrates the importance of carefully planning the best optical configuration to achieve efficient harmonic rejection and obtain correct and reliable XAFS spectra. Here we have illustrated the effect of insufficient harmonic rejection on a thin homogeneous metallic foil, often used as reference sample for EXAFS. However, as already mentioned above, the effects on ‘real’ samples can be much more difficult to detect. Therefore, it is good practice to assure a degree of harmonic rejection that will allow to work safely on any kind of sample. The generally accepted level of rejection is to achieve a ratio of 10^−5^ or less in the flux of photons at the energy of the third harmonic with respect to the flux of photons at the fundamental energy. Detuning to this extent by the monochromator is not feasible. Therefore a pair of harmonic rejection mirrors, with suitable choice of coatings and operational grazing angles, is mandatory. Information on the amount of harmonic rejection by different mirror coatings/substrates are given by Henke *et al.* (1993 ▸). For guidelines to reduce harmonic content, see Tran *et al.* (2003 ▸) and Glover & Chantler (2009 ▸).

### 2.3. Non-compensated monochromator glitches   

The occurrence of multiple reflections within a monochromator crystal gives rise to sharp dips or spikes in the intensity of the diffracted X-ray beam at specific energies. When collecting XAS data, these so-called *monochromator glitches* are usually removed from the spectra by normalizing the signal transmitted by the sample to the incoming beam intensity. Unfortunately, this approach does not always work. Reasons for this include the presence of harmonics in the beam, inhomogeneity and non-uniformity of the sample thickness, and the non-linearity of the two detectors used in the measurements (Stern & Lu, 1982 ▸; Bridges *et al.*, 1992 ▸; Li *et al.*, 1994 ▸).

**Figure 3:**
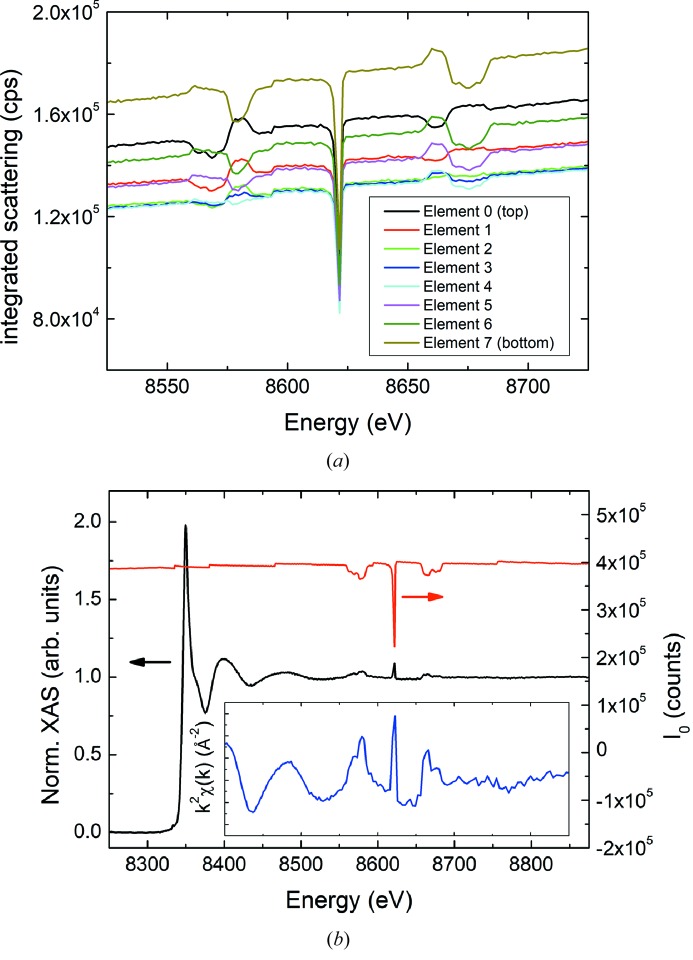
(*a*) Integrated scattering contribution collected by the eight elements at the downstream row of a 64-element fluorescence detector. (*b*) Comparison of the fluorescence Ni *K*-edge XAS and EXAFS signal of a 100 µ*M* nickel nitrate aqueous solution with the incoming intensity collected by the ionization chamber placed before the sample (*I*_0_).

Additionally, it has been recently found that, when collecting the fluorescence signal for very dilute samples using a large-area multi-element detector, these glitches do not compensate (Sutter *et al.*, 2016 ▸). This occurs even after carefully tackling all the experimental issues mentioned above. A detailed investigation of the scattering contributions revealed that this effect is due to changes in the spatial distribution of the quasi-elastically scattered photons over the detector when passing through a glitch. As shown in Fig. 3(*a*) ▸, the intensity drop of the integrated scattering strongly depends on the vertical position of the element in the detector. In very dilute samples, this contribution is significant and cannot be completely separated from the fluorescence signal, thus preventing proper normalization of the glitch features in the spectral data (see Fig. 3*b* ▸).

A fitting procedure treating coherent and Compton scattering developed by Sutter *et al.* (2016 ▸) has shown that the spatial distribution of the quasi-elastically scattered intensity induces changes in the polarization of the incident beam on the sample as a monochromator glitch is traversed. In particular, when the reciprocal lattice vectors of the extra reflections do not lie in the scattering plane of the main diffracted beam, the coupling between the incident and the diffracted beams within the crystal induces a change in the polarization (mainly a rotation of the polarization). This result has been corroborated by multiple-beam dynamical diffraction theory calculations. Post-processing routines to compensate the glitches in XAS data could be developed that include corrections due to these effects.

Although the glitches are an intrinsic phenomenon of the monochromatization process and cannot be avoided, the azimuthal angle of the monochromator crystals can be carefully optimized to minimize the number of glitches appearing within the working energy range. In that respect, some efforts have been made to predict the best configuration for each type of crystal (van der Laan & Thole, 1988 ▸; Tang *et al.*, 2015 ▸).

## 3. Related methods: limitations and recommendations   

### 3.1. Photon-in/photon-out spectroscopy   

Photon-in/photon-out spectroscopy may be defined for an experimental setup that enables measurement of incoming and emitted (scattered) photons with an energy bandwidth of the order of the core-hole lifetime broadening. This distinguishes it from fluorescence-detected absorption spectroscopy using conventional solid state detectors with energy bandwidth approximately two orders of magnitude larger than the core-hole lifetime broadening. High-energy-resolution fluorescence-detected (HERFD) X-ray absorption spectroscopy provides sharper spectral features but *a priori* does not record a spectrum that is proportional to the photoelectric absorption cross section (Carra *et al.*, 1995 ▸). Excitations into strongly localized orbitals (pre-edges) may show strong deviations from the absorption coefficient in HERFD-XAS while excitations into delocalized orbitals (bands that give rise to the main edge) may appear in HERFD-XAS as an absorption spectrum with increased spectral resolution (Glatzel *et al.*, 2013 ▸).

**Figure 4:**
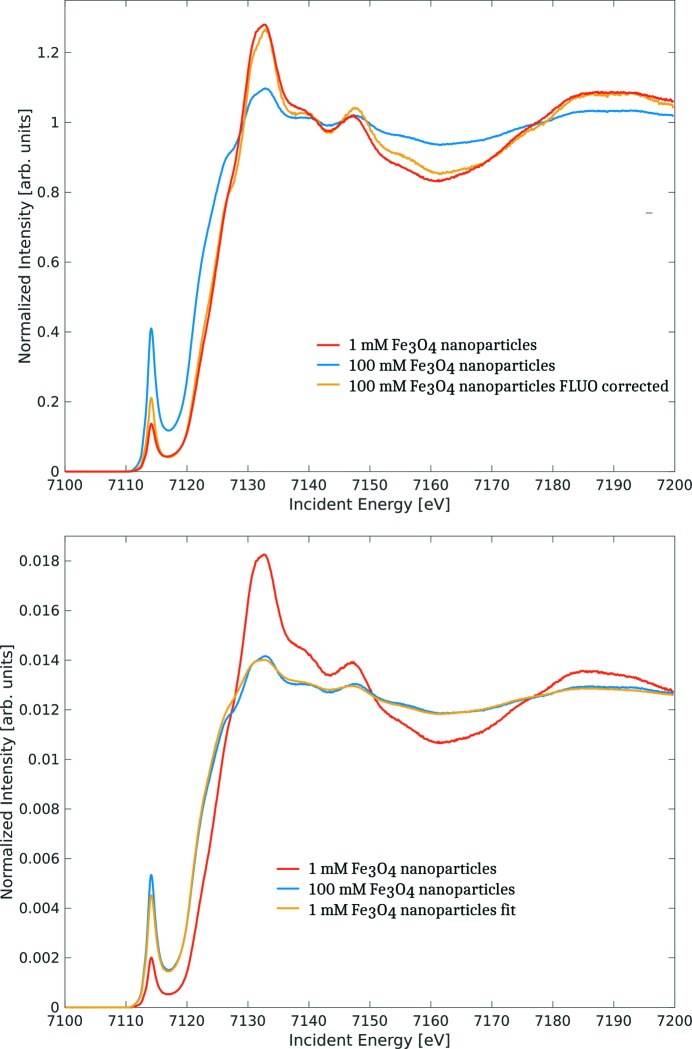
The *K*-edge *K*_α1_-detected HERFD-XANES of Fe in magnetite nano-particles. The self- or over-absorption in the spectrum with 100 m*M* concentration of Fe is clearly visible. The spectral distortion is corrected using the *FLUO* code in the top panel and the strongly distorted spectrum is fitted using the less-distorted spectrum in the bottom panel. Note that the fitted spectra are normalized to the spectral area and not the edge jump.

HERFD-XAS is always distorted by over-absorption (or incident beam self-absorption) just as standard fluorescence-detected absorption spectroscopy (Bunker, 2010 ▸). In many cases, this distortion cannot be corrected [using, for example, the *FLUO* code (Haskel, 1999 ▸)] because the sample composition and other experimental parameters are not sufficiently well defined. In order to understand whether a spectral change arises from this experimental artefact or a real change of the absorption coefficient, we propose to fit the spectra using the general formula for fluorescence-detected absorption spectroscopy (Fig. 4 ▸) (Bianchini & Glatzel, 2012 ▸). In this case, instead of correcting the distortion it is reproduced using fitting parameters. While this procedure does not provide the correct spectrum, it may help to identify the cause of a spectral change. We note that the correction and fitting shown in Fig. 4 ▸ can likely be improved by using a formalism that treats the angles of the incoming and outgoing beam in a more sophisticated fashion.

Some more remarks:

(i) A HERFD-XAS spectrum always requires the chosen emission energy to be reported. As the emission energy may show a chemical dependence, it must be mentioned whether the emission energy was kept constant or changed to stay on the maximum intensity.

(ii) The full captured scattering angle must always be reported as it is an important parameter for photon-in/photon-out experiments.

(iii) The scattering cross section for the different fluorescence lines varies by more than three orders of magnitude. The required dynamic range must be considered for detector linearity. Higher harmonics in the incident beam may give background arising from strong fluorescence lines in the energy range of a weak line of interest.

(iv) X-ray emission spectroscopy always depends on the excitation energy even when chosen well above the absorption edge because of the onset of multiple electron excitations (*e.g.* *KL*-edge) (Glatzel *et al.*, 2003 ▸) and background from Compton scattering.

(v) The instrumental energy bandwidth should be reported. This requires some care as the elastic scattering has a different angular dependence than the inelastic process that is recorded in the actual experiment. In the case of a multi-analyzer crystal instrument, each analyzer is likely to have a different bandwidth because of the limited reproducibility of the manufacturing process.

### 3.2. Energy-dispersive XAS   

The energy-dispersive spectrometer employs a curved crystal to disperse and focus a polychromatic fan of X-rays onto the sample (Matsushita & Phizackerley, 1981 ▸). The transmitted beam is detected by a position-sensitive detector where energy is correlated to position. Besides following time-dependent phenomena (Mathon *et al.*, 2016 ▸), these spectrometers can be used for very specific XAS applications (Pascarelli & Mathon, 2010 ▸, and references therein; Torchio *et al.*, 2016 ▸; Ihli *et al.*, 2017 ▸).

The energy bandwidth Δ*E* diffracted by the crystal is proportional to the Bragg angle variation along the beam footprint, multiplied by the cotangent of the Bragg angle. This leads to the first limitation due to a reduced Δ*E* at low energies: often only XANES is acquired at *E* < 7 keV.

However, the most important limitation of EDXAS is linked to having a polychromatic beam on the sample, so photon-out spectroscopies are not applicable.

Another intrinsic limitation stems from the fact that the XAS spectrum is acquired as a one-dimensional image on the position-sensitive detector. In particular, the energy-direction correlation established at the polychromator must be preserved all the way to the detector, which poses restrictions on sample microstructure to avoid scattering (*i.e.* SAXS), which destroys energy resolution and may introduce artefacts in the spectra. This scattering can be eliminated by using a filter (Hagelstein *et al.*, 1998 ▸), or parallel detection is given up and a monochromatic beam, selected by a slit on the polychromatic fan, is used to measure the spectrum in a step-by-step fashion (Pascarelli *et al.*, 1999 ▸).

**Figure 5:**
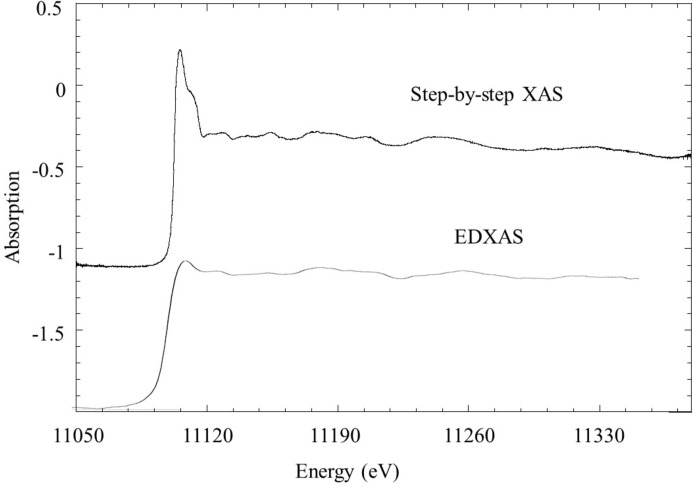
Transmission Ge *K*-edge XAS on a pellet containing Ge micrometer-sized powder in a boron nitride matrix in a 1:10 ratio. Top: step-by-step acquisition using the Turbo XAS variant of EDXAS (Pascarelli *et al.*, 1999 ▸). Bottom: parallel acquisition using a position-sensitive detector in energy-dispersive mode.

The typical EXAFS pellet, for example, is very difficult to handle. Fig. 5 ▸ illustrates an example of a pellet containing Ge micrometer-sized powder in a boron nitride (BN) matrix in a 1:10 ratio. Data have been acquired in transmission mode, using both the step-by-step variant of EDXAS (Pascarelli *et al.*, 1999 ▸) and ion chambers to measure *I*_0_ and *I*_1_, and in the conventional EDXAS mode, with parallel acquisition of the full spectrum on the position-sensitive detector. The figure illustrates that the latter data are affected by strong broadening of the XANES features, due to small-angle scattering from the sample which perturbs the energy–direction correlation established at the polychromator. This scattering is often due to the matrix (*i.e.* BN) of the pellet. Heterogeneous catalysts are another class of very challenging samples, often because of diffusion from the porous microstructure of the support (*i.e.* zeolite).

Another related problem is the high sensitivity to defects in optical elements and samples, enhanced by the increasing coherence length of X-rays from lower-emittance storage rings. In particular, the surface quality of the polychromator crystal plays a major role. Finally, the method is very sensitive to beam instabilities, especially if *I*_0_ and *I*_1_ are not measured simultaneously, but the great advances in source stability nowadays make this a minor problem.

The advent of lower-emittance storage rings will bring additional challenges to EDXAS. Since the spectrum is acquired as a one-dimensional image, the larger horizontal coherence length and its negative effects on the spatial homogeneity of the beam will affect EDXAS to a greater extent than other XAS-based methods. Also, the reduction in horizontal source size will not translate to smaller horizontal spot sizes at the facilities which are already today at the diffraction limit [such as ID24 at the ESRF (Pascarelli *et al.*, 2016 ▸)]. On the other hand, the sharper energy spectrum of undulator emission is not expected to limit the effective energy bandwidth of the data, which will likely remain limited by the quality of the polychromator crystals. Diffraction-limited storage rings are expected to boost applications that exploit the unique capacity of EDXAS to acquire a full XAS spectrum in a single shot, where the total number of photons per X-ray bunch, more than brilliance, is the relevant parameter.

## 4. Increasingly common applications   

### 4.1. High-pressure XAS   

Matter undergoes changes in its physical, chemical and structural characteristics when subject to high pressures. Under pressure, atoms are forced closer in a smaller volume and the energy of the atomic bonds is changed. In addition, pressure serves as a tool for synthesizing new materials and is especially important in the study of the rocks and minerals constituting the interior of the Earth and other planets. High-pressure research has been progressing rapidly in the last decades thanks to the concomitant development of high-pressure technology and to the increased accuracy of probing methods (Mao *et al.*, 2016 ▸). Synchrotron sources offer now a large portfolio of techniques that can be applied to understand a material’s behaviour under extreme environment from all aspects. X-ray absorption spectroscopy is one of these techniques. Diamond anvil cells (DACs) are the most common device for pressure generation (Eremets, 1996 ▸) and are widely used for XAS at high pressure. They can generate static pressure conditions above 750 GPa (Dubrovinsky *et al.*, 2015 ▸). The concept of a DAC consists of forcing together two flawless diamonds against a microgram-sized sample (Eremets, 1996 ▸).

**Figure 6:**
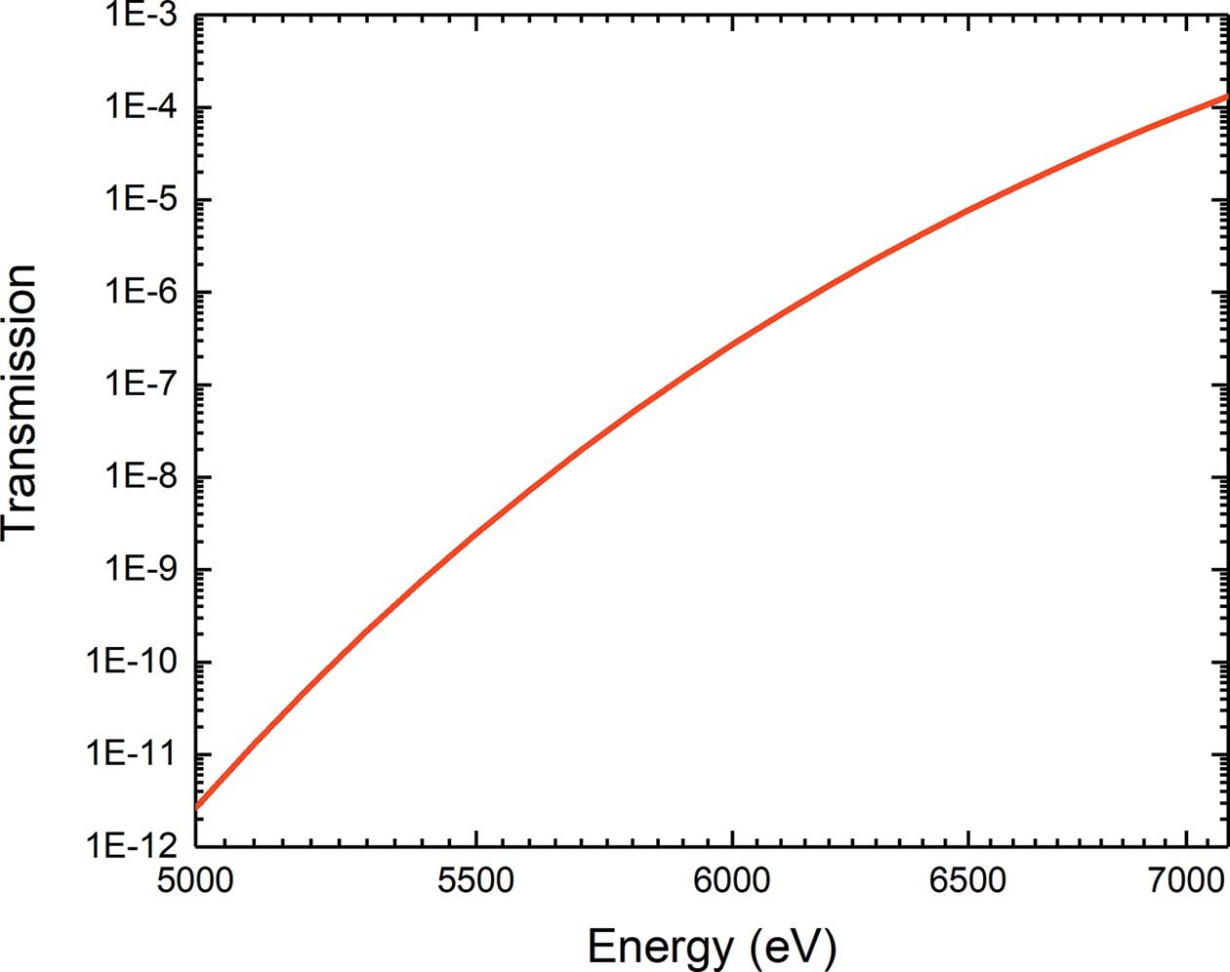
Transmission of diamond of 4 mm thickness between 5.0 and 7.0 keV.

An issue related to the use of DAC for XAS regards the high absorption of the diamond anvils at energies below 7 keV. The transmission of 4 mm of diamonds, corresponding to the total thickness of a couple of standard anvils, is shown in Fig. 6 ▸. The main solution to this issue is to reduce the thickness of the diamonds using a combination of fully perforated diamond as diamond backing plate with a miniature anvil and a partially perforated diamond. In this way the diamond thickness along the X-ray path can be reduced down to less than 1 mm maintaining at the same time a good mechanical strength to reach pressures in excess of 100 GPa (Dadashev *et al.*, 2001 ▸). As an alternative, the DAC can be oriented so that X-rays travel through a low-*Z* gasket such as Be (Itié *et al.*, 2007 ▸). The fluorescence signal is collected at 90° with respect to the incident beam. Besides safety issues related to machining of Be, the main drawback of this geometry is that the beam path through the gasket changes with pressure.

The other and principal technical issue related to the use of DACs for XAS is the crystal structure of the diamond anvils. In fact, in the large energy range in which the absorption coefficient is measured, Bragg conditions are satisfied for the crystal structure of the diamond causing the X-rays to be diffracted by the anvils. This removes photons at precise energy values leading to large dips in the transmitted X-ray intensity, appearing as large peaks in the spectra. The ultimate solution to this is to use nano-polycrystalline diamond (NPD) anvils instead of single-crystal diamond anvils (Irifune *et al.*, 2003 ▸; Ishimatsu *et al.*, 2012 ▸). These consist of randomly oriented single-phase diamond grains of several tens of nanometers. Since the grains are randomly oriented, Bragg’s law is always satisfied independently of the energy, therefore the intensity of the diffracted X-ray changes moderately with energy and consequently the NPD imparts a smooth background to the absorption profile.

### 4.2. Transient XAS studies of catalysts   

Using hard X-ray (>4.5 keV) based techniques, such as XAS, to study catalytic reaction mechanisms has a major advantage that catalytic materials can be studied under operating conditions, *i.e.* in a catalytic reactor. Such studies have been made since the early 1980s at synchrotron light sources and have led to an understanding of structure–performance relationships of many heterogeneous catalytic systems (Bare & Ressler, 2009 ▸). Many such XAS studies (at least on the higher weight loaded samples) can nowadays potentially be carried out using a commercial laboratory-based X-ray spectrometer.

To rationally design and improve catalytic processes, an understanding of the reaction mechanism including reaction intermediates is essential. In a seminal paper, Oyama *et al.* (Bravo-Suárez *et al.*, 2008 ▸) showed that the observation of a specific structure by EXAFS under operating conditions does not necessarily mean that this structure is involved in the catalytic reaction mechanism. This structure, resembling a particular structural site, might well be a spectator species to the catalytic reaction. Transient X-ray spectroscopy, which can only be performed at a synchrotron, allows the rate of structural change to be measured. When the rate of structural change matches the rate of the overall reaction as measured for example using a mass spectrometer, then this structure, or alternatively called active site, is involved in the rate-limiting step.

The question then arises, what time resolution is needed to decipher the rate-limiting step in a heterogeneous catalytic reaction? When one considers the rates of important industrial catalytic processes, one will observe that these rates rarely exceed 10 ms. For example, the rate of ammonia synthesis is of the order of a second (Nørskov *et al.*, 2009 ▸). As a consequence, the spectroscopic technique employed needs to have an ultimate time resolution of a few milliseconds to catch the species involved in the rate-limiting step. Time-resolved XAS with this time resolution can for example be obtained with energy-dispersive XAS (§3.2) or with the quick-EXAFS technique (Müller *et al.*, 2016 ▸). Both techniques can be applied for *in situ* studies in transmission mode, whereas only quick-EXAFS can be combined with fluorescence/emission detection, by using for example a PIPS diode. In the case of quick-EXAFS, the time resolution that can ultimately be achieved is not limited by the mechanics/driving mechanism of the monochromator system but rather by the response time of the detectors used. When one considers that the collection of an EXAFS spectrum takes about 10 ms, and that such an EXAFS spectrum consists of 1000 eV where individual data points need to be 1 eV apart, one sees that the response time of the detectors needs to be at least 10 ms per 1000 energy points = 10 µs per energy point. PIPS detectors, used for the detection of the fluorescence, typically have a sub-microsecond rise-time, whereas ionization chambers, used for the detection of the incoming and transmitted X-rays, are limited by the drift time of the ions and typically have a response time of hundreds of microseconds. Gridded ionization chambers, where a grid is placed between the high-voltage plate and the ground, have a rise time of a few microseconds (Müller *et al.*, 2013 ▸) and overcome this limitation. The next step to be taken is to improve the response time of the current amplifier, which is on the order of 5–10 µs for gains of 6 and 7.

When one uses a dispersive crystal spectrometer to detect X-ray emission spectra with a resolution of around 1 eV in a single shot (Kopelent *et al.*, 2016 ▸), at (fixed incident) energies just before the X-ray absorption edge, then time-resolved resonant X-ray emission spectroscopy can be performed with sub-second time resolution and has a high sensitivity to small changes. This technique was recently used to determine small changes in the redox state of Ce during CO oxidation (Kopelent *et al.*, 2015 ▸). Active Ce^3+^ species in a ceria-supported platinum catalyst during CO oxidation are short-lived and therefore cannot be observed under steady-state conditions. Using resonant X-ray emission spectroscopy it could be shown that the initial rate of Ce^3+^ formation under transient conditions is quantitatively correlated to the overall rate of CO oxidation under steady-state conditions and thus that ceria reduction is a kinetically relevant step in CO oxidation, whereas a fraction of Ce^3+^ was present as spectators.

## 5. Conclusions and outlook   

We report here a summary of the problems discussed at the Q2XAFS Workshop and Satellite to IUCr Congress 2017 on different aspects of a XAFS experiment that affect data quality. The aim is to maintain the larger XAFS community informed on the outcome of the meeting, and not to give a comprehensive overview of the field. From the selection of topics reported here, we see that a few outstanding problems persist and ways to overcome some of them are addressed. Novel developments in methods or in the field of two increasingly common applications are presented, coupled to good practice recommendations.

In the coming years, with the arrival on the synchrotron scene of diffraction-limited storage rings, and with the high-energy free-electron lasers coming into full operation, our XAFS community will be confronted with important challenges. The continuing growth of a strong XAFS community will rely more and more on our ability to maintain close communication between ‘method developers’ and inexperienced users, which, given the multidisciplinary nature of the technique, are expected to span a wider and wider horizon of different scientific domains. In this context, implementing a more robust strategy for the application of XAFS standards and criteria will be increasingly important and desirable.

## Footnotes

1 The Q2XAFS acronym was coined at the First International Workshop on ‘Improving data Quality and Quantity for XAFS experiments’, that took place on 8–9 December 2011 at Photon Factory, Tsukuba, Japan.
